# Association Between the CALLY Index and Carotid Artery Stenosis Severity in Patients Younger and Older than 65 Years

**DOI:** 10.3390/jcm15114242

**Published:** 2026-05-30

**Authors:** Demet Doğan, Erce Güler

**Affiliations:** Department of Radiology, Faculty of Medicine, İstanbul Okan University, İstanbul 34947, Türkiye; erceguler@hotmail.com

**Keywords:** carotid stenosis, aged, C-reactive protein, Albumins, Lymphocytes

## Abstract

**Background/Objectives**: Atherosclerosis is a multifaceted inflammatory condition closely linked to immunonutritional status. The C-reactive protein–albumin–lymphocyte (CALLY) index is a composite marker of immunonutrition. We assessed the relationship between this index, age, and carotid stenosis in individuals undergoing carotid Doppler ultrasonography. **Methods**: Individuals who underwent routine carotid Doppler ultrasonography were retrospectively analyzed. Carotid stenosis was categorized by severity, and bilateral intima–media thickness was recorded. The composite index integrating C-reactive protein, albumin, and lymphocyte parameters was derived from laboratory parameters. Traditional cardiovascular risk factors, including hypertension, diabetes mellitus, hyperlipidemia, and smoking status, were also recorded and incorporated into multivariable analyses. Sex-related differences were additionally evaluated using sex-stratified correlation analyses. **Results**: Among 1516 patients, 895 (59.1%) were younger than 65 years and 621 (40.9%) were aged 65 years and above. Carotid artery stenosis and intima–media thickness were significantly higher in individuals aged 65 years and above. The mean index value was significantly lower in the older group (4.0 versus 8.3; *p* < 0.001). Hypertension, diabetes mellitus, and hyperlipidemia were significantly more prevalent in patients with carotid stenosis (*p* < 0.001 for all), whereas smoking was not associated (*p* > 0.05). Male sex was independently associated with carotid stenosis, and sex-stratified analyses demonstrated similar inverse associations between age and CALLY index values in both sexes. In multivariable analysis, lower index values were independently associated with carotid stenosis and carotid intima–media thickness in both age groups. In the overall cohort, the index demonstrated moderate discriminatory performance (area under the curve = 0.724). Similar moderate performance was observed in the <65 (AUC = 0.681) and ≥65 (AUC = 0.685) subgroups. **Conclusions**: The CALLY index was independently associated with carotid stenosis and CIMT after adjustment for traditional cardiovascular risk factors. Although effect sizes were comparable between age groups, CALLY index levels were lower in older individuals. These findings suggest that the CALLY index may provide complementary information in the assessment of carotid atherosclerosis, particularly in elderly populations. However, given the retrospective cross-sectional design and moderate discriminatory performance, it should not be interpreted as a standalone, causal, or disease-specific marker.

## 1. Introduction

Carotid artery stenosis, a significant clinical manifestation of atherosclerosis, is a key factor in the risk of cerebrovascular events [1]. Doppler ultrasonography (US) is a non-invasive, safe, low-cost, and easily applicable imaging method used in the evaluation of carotid vasculature. Measurement of carotid intima–media thickness (CIMT) during Doppler US examination is useful in detecting early subclinical signs of atherosclerosis [2,3]. However, the degree of carotid stenosis, primarily assessed by Doppler US, is a significant factor in terms of symptomatic cerebrovascular events and long-term prognosis, and plays a decisive role in clinical decision-making for medical, endovascular, or surgical treatment [1,3].

Recently, interest has shifted toward composite biomarkers that simultaneously reflect systemic inflammatory activity and nutritional reserve. The CALLY index, derived from C-reactive protein (CRP), albumin, and lymphocyte values, integrates these immunonutritional dimensions into a single measure. Although initially used as a prognostic indicator in oncology [4,5], the inverse association of the index with adverse cardiovascular outcomes has increasingly been recognized [6,7,8]. However, whether the CALLY index provides information beyond traditional cardiovascular risk factors such as hypertension, diabetes mellitus, and hyperlipidemia remains unclear.

The aging process introduces “inflammaging,” characterized by persistent, mild systemic inflammatory activity, alongside dysregulated immune responses and heightened nutritional vulnerability. Circulating inflammatory markers tend to increase with advancing age. These biological alterations play a significant role in the development and progression of atherosclerotic disease [9,10]. Consequently, immunonutritional indices may hold greater clinical relevance in older adults. In geriatric populations, vascular aging rarely occurs in isolation but is frequently accompanied by frailty, sarcopenia, multimorbidity, polypharmacy, and functional disability. These geriatric conditions may influence both systemic inflammatory activity and nutritional reserve, thereby affecting CALLY index components such as CRP, albumin, and lymphocyte count. Frailty and sarcopenia are particularly relevant because they are associated with chronic low-grade inflammation, reduced protein reserve, impaired immune regulation, and adverse cardiovascular outcomes. Similarly, multimorbidity and polypharmacy may modify inflammatory and nutritional biomarkers and complicate the interpretation of composite indices in older adults. Therefore, these geriatric syndromes should be considered as important contextual factors when interpreting the relationship between the CALLY index and carotid atherosclerotic burden in older adults. Since the CALLY index incorporates inflammatory and nutritional parameters, its association with carotid atherosclerotic burden may vary across different age groups. In addition, sex-related differences in inflammatory activity, immune regulation, and cardiovascular risk profiles may also influence the relationship between immunonutritional indices and atherosclerotic burden.

Although the CALLY index has been associated with outcomes in various cardiovascular diseases, studies evaluating its association with carotid atherosclerosis in particular are limited. Current data are generally obtained from highly selective populations, and there is a gap in research on its relationship with key structural indicators such as CIMT and stenosis severity, and with age, in routine clinical practice. The aim of this study was to investigate the statistical association between the CALLY index and carotid atherosclerosis-related imaging findings, including carotid artery stenosis, stenosis severity, and carotid intima–media thickness (CIMT), in individuals undergoing routine carotid Doppler ultrasonography, and to determine whether these associations differ according to age, while accounting for established cardiovascular risk factors.

## 2. Materials and Methods

### 2.1. Ethical Approval

This retrospective study was conducted in accordance with the rules set forth in the “Helsinki Declaration”, “Guidelines for Good Medical Practice” and “Guidelines for Good Laboratory Practice”. Approval for the study was obtained from the Istanbul Okan University Ethics Committee (Decision No: 194/10 Date: 5 November 2025). Informed consent was not deemed necessary as only imaging and laboratory data were used. AI-assisted language editing was used to improve grammar, clarity, and readability; all scientific content, analyses, and final wording were reviewed and approved by the authors.

### 2.2. Study Design

Data from adult individuals who underwent carotid Doppler ultrasonography at a tertiary university hospital between September 2019 and November 2025 were retrospectively analyzed. Eligibility was restricted to patients aged 18 years or older with available carotid Doppler US data and concurrent laboratory results. To ensure temporal convergence between laboratory data and imaging findings and to maintain adequate sample size, a time interval of ±10 days was chosen. Adult individuals in whom the necessary laboratory parameters (CRP, albumin, and lymphocyte values) for calculating the CALLY index were available within this period were included in the study. Individuals with missing laboratory parameters required for calculation of the CALLY index, missing carotid Doppler ultrasonography data, unavailable clinical covariates included in multivariable models, poor ultrasonographic image quality, or documented active infection or malignancy at the time of evaluation were excluded from the study. Therefore, the final analyses were conducted using a complete-case cohort. No statistical imputation was performed. Active infection was defined based on documented clinical diagnoses and/or ongoing anti-infective treatment recorded in the institutional electronic health system. Additionally, patients with laboratory findings suggestive of acute infection or inflammation, such as leukocytosis and neutrophilia, were excluded from the study. This approach was adopted to minimize potential confounding effects on inflammatory and immunonutritional parameters. For comparative analysis, the final cohort was stratified into two age-based categories: individuals under 65 years and those 65 years and above.

### 2.3. Data Collection

Demographic, laboratory, and imaging data were obtained from the institutional electronic database (Nucleus) in addition to the Picture Archiving and Communication System (PACS). The CALLY index was computed as follows: CALLY index = (albumin × lymphocyte count)/CRP (Albumin in g/dL, lymphocyte count in 10^9^/L, and CRP in mg/L) consistent with the original formulation reported in the literature, without any additional scaling factors.

### 2.4. Ultrasound Evaluation

Carotid Doppler US was performed by radiologists working in the Radiology Department on a rotational basis. Examinations were conducted using the GE Logiq S7 Expert, GE Logiq S8 Expert, and GE Logiq P7 ultrasound systems (GE Healthcare, Chicago, IL, USA) equipped with high-resolution linear transducers (6–9 MHz). In each case, grayscale images were obtained in longitudinal and transverse planes; additionally, color and spectral Doppler flow measurements were performed encompassing the common carotid artery, the bifurcation region, and the internal carotid artery. CIMT measurements were performed bilaterally at the visually identified thickest segment of the distal common carotid artery along the posterior wall. Measurements were performed on longitudinal B-mode images, and the thickest segments visualized on both the right and left sides were recorded separately. All measurements were performed in accordance with current guideline recommendations and by experienced radiologists in vascular ultrasonography. To reduce measurement variability, standardized imaging protocols were applied across all examinations. Because the study was retrospective and carotid Doppler ultrasonography examinations were performed as part of routine clinical practice by radiologists on a rotational basis, a formal interobserver variability analysis was not performed. Nevertheless, standardized departmental imaging protocols were used to minimize measurement variability, including bilateral CIMT assessment on longitudinal B-mode images and stenosis grading based on grayscale, color Doppler, and spectral Doppler criteria. Carotid artery stenosis was evaluated using the grayscale ultrasound findings along with color and spectral Doppler velocity criteria. The severity of carotid stenosis was determined according to the NASCET methodology. Less than 50%, 50–70%, and more than 70% were used to represent mild, moderate, and severe stenosis, respectively, and a normal category and occlusion was also defined.

### 2.5. Statistical Analysis

Descriptive statistics were summarized using measures of central tendency and dispersion together with frequency and percentage distributions. Normality of data distribution was evaluated using the Kolmogorov–Smirnov and Shapiro–Wilk tests. Comparisons involving non-normally distributed continuous variables were performed using the Mann–Whitney U test. Associations between categorical variables were examined with the Chi-square test. Comparisons across multiple stenosis severity categories were performed using the Kruskal–Wallis test, followed by post hoc pairwise analyses where appropriate. Correlations between continuous variables, including associations of the CALLY index with age and carotid intima–media thickness (CIMT), were assessed using Pearson and Spearman correlation analyses, including sex-stratified subgroup analyses where appropriate. Effect size estimation and determination of optimal threshold values were conducted using receiver operating characteristic (ROC) curve analysis. Optimal cutoff values were determined using the Youden index. To evaluate independent associations, multivariable logistic regression models were constructed including age, sex, hypertension, diabetes mellitus, hyperlipidemia, smoking status, and the CALLY index. Standardized effect size analysis was performed using z-score normalization of the CALLY index. Additionally, multivariable linear regression analyses were performed to assess the independent relationship between the CALLY index and CIMT measurements. All statistical procedures were carried out using IBM SPSS Statistics software (version 27.0; IBM Corp., Armonk, NY, USA).

## 3. Results

### 3.1. Comparison Between the Under-65 and 65-and-Above Groups

The study comprised 1516 patients. Among them, 895 (59.1%) were under 65 years of age, whereas 621 (40.9%) were 65 years and above. Sex distribution did not differ significantly across age categories (*p* > 0.05). Older adults exhibited significantly higher carotid intima–media thickness (CIMT) across left and right measurements compared to the younger cohort (*p* < 0.05 for all comparisons). Furthermore, carotid artery stenosis occurred more frequently in the ≥65-year group (*p* < 0.05). Laboratory analysis revealed that serum albumin and lymphocyte concentrations were notably reduced among older individuals, while CRP concentrations were markedly elevated (*p* < 0.05 for all comparisons). Consequently, CALLY index values were reduced among older individuals (median 2.1 vs. 6.0; *p* < 0.05 for comparison). The mean value of the CALLY score showed a similar pattern, measuring 4.0 in the ≥65-year group compared with 8.3 in the under-65 group (*p* < 0.001). Regarding clinical pathways, neurology referrals were more prevalent among younger patients, whereas referrals from cardiovascular surgery and other departments were more frequent among older patients (*p* < 0.05 for all comparisons; Table 1).

Correlation analyses demonstrated a significant inverse association between age and CALLY index values in the overall cohort (Pearson r = −0.308, Spearman r = −0.376; *p* < 0.001 for both analyses). Sex-stratified analyses showed similar inverse correlations in both men (Pearson r = −0.329, Spearman r = −0.411; *p* < 0.001 for both) and women (Pearson r = −0.279, Spearman r = −0.327; *p* < 0.001 for both), although the correlation appeared slightly stronger in men.

### 3.2. Relationship of the CALLY Index with Carotid Stenosis in the Under-65 Group

Within the younger cohort, individuals with carotid artery stenosis were older and more frequently male compared with those without stenosis (both *p* < 0.05). CIMT measurements were higher in the stenosis group across all parameters (*p* < 0.05 for all comparisons).

Regarding laboratory findings, albumin concentrations were lower and CRP concentrations higher among younger individuals with stenosis (both *p* < 0.05), whereas lymphocyte counts remained comparable (*p* > 0.05). Critically, CALLY index values appeared lower in younger individuals diagnosed with stenosis (*p* < 0.05; Table 2).

Receiver operating characteristic analysis showed that CALLY index values had moderate ability to distinguish stenosis in this age category (AUC = 0.681; 95% CI: 0.642–0.715; *p* < 0.001). With a cutoff of 4.0, sensitivity was 70.3% and specificity 60.8%.

### 3.3. Relationship of the CALLY Index with Carotid Stenosis in the 65-and-Above Group

Similarly, within the ≥65-year category, individuals with stenosis tended to be older and were more often male (*p* < 0.05 for both comparisons). CIMT values were higher across all parameters in those with stenosis (*p* < 0.05 for all comparisons).

In terms of laboratory markers, albumin and lymphocyte concentrations were reduced, whereas CRP concentrations were elevated in older individuals with stenosis (*p* < 0.05 for all comparisons). CALLY index values were lower in this age category in the presence of carotid stenosis (*p* < 0.05; Table 3).

Receiver operating characteristic analysis showed moderate discriminative performance in the ≥65-year group (AUC = 0.685; 95% CI: 0.632–0.739; *p* < 0.001). With an optimal cutoff of 2.3, sensitivity was 70.5% and specificity 56.7%.

### 3.4. Association Between the CALLY Index and Stenosis Severity

When patients were stratified according to stenosis severity (normal, mild, moderate, severe, and occlusion), carotid intima–media thickness (CIMT) values increased progressively across categories (*p* < 0.001, Kruskal–Wallis test). Conversely, CALLY index values demonstrated a stepwise decline with increasing stenosis severity (*p* < 0.001, Kruskal–Wallis test). Median CALLY values decreased from 6.62 in individuals without stenosis to 0.67 in those with occlusion. Pairwise comparisons confirmed that patients with moderate, severe, and occlusive stenosis exhibited significantly lower CALLY index values compared with the normal group. These findings indicate a graded inverse relationship between CALLY index levels and carotid artery stenosis severity (Table 4).

In addition, the prevalence of hypertension, diabetes mellitus, and hyperlipidemia increased significantly with stenosis severity (*p* < 0.001), whereas smoking status showed no significant association (*p* > 0.05). Similarly, carotid intima–media thickness values were significantly higher in individuals with hypertension, diabetes mellitus, and hyperlipidemia, as well as in older and male patients, whereas no significant association was observed with smoking status (Table 5).

### 3.5. Association of Baseline Characteristics with Carotid Stenosis

Hypertension, diabetes mellitus, and hyperlipidemia were significantly more prevalent in patients with carotid stenosis compared to those without stenosis (*p* < 0.001 for all). In contrast, smoking status did not differ significantly between groups (*p* = 0.680) (Table 6).

### 3.6. Multivariable Logistic Regression Analysis

Multivariable logistic regression analyses were performed separately for the overall cohort and for the age-based subgroups (<65 years and ≥65 years), including age, sex, hypertension, diabetes mellitus, hyperlipidemia, smoking status, and the CALLY index. In all models, the CALLY index remained independently associated with carotid stenosis (overall cohort: OR 0.943, 95% CI 0.923–0.964, *p* < 0.001; age < 65 years: OR 0.942, 95% CI 0.918–0.967, *p* < 0.001; age ≥ 65 years: OR 0.939, 95% CI 0.904–0.975, *p* = 0.001) (Table 7, Table 8 and Table 9).

To improve clinical interpretability, an additional standardized effect size analysis was performed using the z-score-transformed CALLY index. In this standardized model, a 1 standard deviation decrease in the CALLY index was associated with approximately a 2.4-fold increase in the odds of carotid stenosis (OR 2.43, 95% CI 2.08–2.85, *p* < 0.001).

Among traditional cardiovascular risk factors, hypertension and hyperlipidemia remained independently associated with carotid stenosis across the regression models, whereas diabetes mellitus and smoking were not significant predictors. When analyses were stratified according to age group, the CALLY index remained significantly associated with carotid stenosis in both the <65-year and ≥65-year subgroups, with comparable effect sizes across models. These findings indicate that the association between the CALLY index and carotid stenosis persists independently of conventional cardiovascular risk factors in both younger and older individuals.

### 3.7. Multivariable Linear Regression Analysis

Multivariable linear regression analysis demonstrated that the CALLY index was independently associated with both left and right CIMT measurements (β = −0.007, *p* < 0.001 for both). Age, male sex, and hypertension were also significantly associated with increased CIMT values. In addition, lymphocyte count and CRP levels showed independent associations, whereas diabetes mellitus and smoking were not significant predictors. These findings indicate that the relationship between the CALLY index and CIMT persists independently of traditional cardiovascular risk factors (Table 10).

## 4. Discussion

Given the complex and multifactorial pathophysiology of cardiovascular diseases, interest in composite indices that simultaneously reflect inflammatory, metabolic, and immunological pathways has increased. Several blood-derived markers, including the triglyceride–glucose (TyG) index, the neutrophil-to-lymphocyte ratio (NLR) and the platelet-to-lymphocyte ratio (PLR), are linked to atherosclerotic burden and unfavorable cardiovascular outcomes [11,12,13]. Markers including troponin T (TnT), C-reactive protein (CRP), N-terminal pro–brain natriuretic peptide (NT-proBNP), together with D-dimer, have been reported to aid in prognostic evaluation of cardiovascular disease [14,15]. These results highlight the contribution of systemic inflammatory activity and metabolic alterations to atherosclerotic progression. Multiple studies have examined the association linking circulating biomarkers to carotid artery disease using Doppler US. In particular, metabolic and inflammation-related indices are associated with carotid atherosclerosis and plaque burden. For example, the TyG index has been reported to relate to the presence as well as the severity of carotid atherosclerotic changes detected by US, suggesting its potential value as an indicator of metabolic and vascular risk [16]. It has been reported that this index, in combination with carotid ultrasonography findings, predicts ischemic stroke and clinical outcomes, while elevated values of this index were observed in this patient group. These findings emphasize the clinical importance of evaluating laboratory-derived biomarkers alongside carotid imaging findings [17,18]. Similarly, previous research suggests that NLR may help identify carotid plaque on ultrasonographic examination [19].

In this context, because the CALLY index incorporates systemic inflammatory, nutritional, and immune-related parameters, it may provide complementary laboratory information in patients evaluated for carotid atherosclerosis. This index may be particularly relevant to investigate in older adults, in whom immunonutritional dysregulation becomes more pronounced. The CALLY index has primarily been investigated in oncological populations [20] and evaluated in some inflammatory diseases [21]. Subsequently, studies examining its association with cardiovascular diseases have been conducted [22,23,24], and reduced CALLY index values have been reported in association with adverse cardiovascular outcomes and arterial disease measures. Nevertheless, evidence regarding the relationship between this index and carotid atherosclerosis remains limited [25]. The main finding of this study is that the CALLY index remained statistically and independently associated with carotid stenosis after adjustment for traditional cardiovascular risk factors, including hypertension, diabetes mellitus, and hyperlipidemia. This suggests that the observed statistical association was not fully explained by conventional cardiovascular risk factors and may support the relevance of immunonutritional status in the assessment of carotid atherosclerosis; however, causality or disease-specific pathophysiological mechanisms cannot be inferred from these data. Consistent with the literature, carotid artery stenosis was more frequent and more severe in elderly individuals in this study. Furthermore, CALLY index values declined progressively with advancing age, and lower CALLY values were associated with more pronounced carotid atherosclerosis-related imaging findings. The lower CALLY index values observed in older individuals may reflect the combined effects of age-related chronic low-grade inflammation (“inflammaging”), immunosenescence, and declining nutritional reserve. Aging is associated with persistent systemic inflammatory activation together with alterations in immune cell regulation and protein metabolism, which may contribute to higher CRP levels, lower albumin concentrations, and reduced lymphocyte counts. These biological changes may promote endothelial dysfunction, vascular inflammation, and progression of atherosclerosis. Therefore, the inverse association observed between the CALLY index and carotid atherosclerosis-related findings in elderly individuals may be interpreted within the broader context of immune-nutritional imbalance and age-related vascular changes. In addition, sex-stratified correlation analyses demonstrated that the inverse relationship between age and CALLY index values was present in both men and women, although the correlation appeared slightly stronger in men. These findings suggest that the age-related decline in CALLY index values is not restricted to a single sex and may be related to broader immunonutritional and inflammatory alterations associated with aging. The relatively stronger correlation observed in men may be related to sex-related differences in cardiovascular risk burden, inflammatory profile, body composition, or hormonal regulation; however, the exact mechanisms remain unclear and require further investigation. These findings suggest that laboratory measures related to systemic inflammation and immunonutritional status may provide complementary information in the evaluation of carotid atherosclerosis. In addition, CALLY index values decreased progressively as stenosis severity increased, while CIMT values showed a parallel rise. This graded pattern suggests that the association is not limited to the presence of stenosis but is also related to its extent. These findings suggest that the CALLY index is a laboratory-derived measure statistically associated with carotid atherosclerosis-related imaging findings. Furthermore, the independent association between the CALLY index and CIMT observed in multivariable linear regression analysis suggests that this statistical relationship was also present for CIMT, an imaging marker of subclinical atherosclerosis. Clinically, the CALLY index is readily derived using standard laboratory measurements and, when interpreted alongside imaging findings such as carotid Doppler ultrasound and CIMT, may provide complementary information in the assessment of carotid atherosclerosis. Although the per-unit effect size of the CALLY index appeared modest in the multivariable regression analysis, standardized effect size analysis demonstrated that a 1 standard deviation decrease in the CALLY index was associated with approximately a 2.4-fold increase in the odds of carotid stenosis. This finding suggests that larger reductions in the CALLY index were statistically associated with the presence of carotid stenosis. Taken together, these findings suggest that the CALLY index may provide complementary, association-based information alongside traditional cardiovascular risk factors and conventional imaging markers in the evaluation of carotid atherosclerosis.

### Strengths and Limitations

This study was conducted in a large population undergoing routine carotid Doppler ultrasonography, increasing the generalizability of the findings to routine clinical practice. Importantly, age-specific assessment was performed by categorizing patients according to their age (<65 and ≥65 years). To our knowledge, studies evaluating the CALLY index across age groups, particularly in elderly patients, are limited; this is a significant strength of the current study. In addition, major cardiovascular risk factors, including hypertension, diabetes mellitus, hyperlipidemia, and smoking status, were incorporated into the multivariable analysis, allowing assessment of whether the association between the CALLY index and carotid stenosis persisted after adjustment for traditional risk determinants. However, several limitations should be acknowledged. First, this was a retrospective, single-center, imaging-based study, which may limit the generalizability of the findings. Furthermore, because of the retrospective cross-sectional design, temporal or causal relationships cannot be established. Therefore, reverse causality cannot be excluded, as established carotid atherosclerosis and the accompanying inflammatory state may have influenced CRP, albumin, lymphocyte count, and consequently CALLY index values, rather than lower CALLY index values necessarily preceding vascular disease. In addition, although standardized ultrasonography protocols were used, formal interobserver variability analysis could not be performed because of the retrospective routine-practice design. Although key cardiovascular risk factors were included, detailed geriatric and clinical variables, including frailty status, functional capacity, body composition or sarcopenia-related measures, medication burden, disease duration, and treatment status, were not available, which may have introduced residual confounding. Therefore, prospective, multicenter studies including more comprehensive clinical and geriatric data are needed to validate and expand upon these findings.

## 5. Conclusions

In this retrospective cross-sectional study, the CALLY index showed an independent statistical association with carotid artery stenosis and stenosis severity after adjustment for traditional cardiovascular risk factors. In addition, its association with carotid intima–media thickness suggests that this statistical relationship was also present for an imaging marker of subclinical atherosclerosis. The CALLY index, derived from routine laboratory parameters, may aid in the assessment of carotid atherosclerosis in daily clinical practice when interpreted alongside carotid Doppler ultrasonography findings. Particularly in elderly populations, where immunonutritional imbalance and chronic low-grade inflammation are more pronounced, it may serve as a simple adjunctive laboratory index for contextual interpretation of carotid atherosclerosis. However, given its moderate diagnostic performance and the retrospective cross-sectional design of the study, the CALLY index should not be used as a standalone tool and should not be interpreted as evidence of predictive superiority, causality, or pathophysiological specificity.

## Figures and Tables

**Table 1:** Clinical, Laboratory, and Ultrasonographic Findings Stratified by Age (<65 vs. ≥65 Years).

		Age < 65 (*n* = 895)				Age ≥ 65 (*n* = 621)				*p*	
		Mean ± SD, *n* (%)			Median	Mean ± SD, *n* (%)			Median		
Sex	Female	348		38.9%		254		40.9%		0.429	^X2^
	Male	547		61.1%		367		59.1%			
Carotid Intima–Media Thickness (mm)											
Left		0.74	±	0.23	0.70	1.08	±	0.24	1.10	0.000	^m^
Right		0.74	±	0.22	0.70	1.05	±	0.24	1.10	0.000	^m^
Degree of Stenosis	Normal	643		71.8%		127		20.5%		0.000	^X2^
	Mild	222		24.8%		374		60.2%			
	Moderate	17		1.9%		70		11.3%			
	Severe	11		1.2%		42		6.8%			
	Occlusion	2		0.2%		8		1.3%			
Albumin		4.2	±	0.6	4.2	3.7	±	0.6	3.8	0.000	^m^
Lymphocyte		2.1	±	0.8	2.1	1.9	±	1.0	1.8	0.000	^m^
CRP		5.0	±	16.7	1.5	7.9	±	18.4	3.0	0.000	^m^
CALLY Index		8.3	±	8.5	6.0	4.0	±	5.1	2.1	0.000	^m^
Department	Neurology	512		57.6%		208		33.9%		0.000	^X2^
	Cardiovascular Surgery	294		33.1%		295		48.1%		0.000	^X2^
	Other	83		9.3%		110		17.9%		0.000	^X2^

^m^ Mann–Whitney U test/^X2^ Chi-square test.

**Table 2:** Characteristics of Individuals Aged <65 Years by Carotid Artery Stenosis Status.

		Stenosis (−) (*n* = 643)				Stenosis (+) (*n* = 252)				*p*	
		Mean ± SD, *n* (%)			Median	Mean ± SD, *n* (%)			Median		
Age		46.7	±	9.6	47.0	56.5	±	6.2	59.0	0.000	^m^
Sex	Female	276		42.9%		72		28.6%		0.000	^X2^
	Male	367		57.1%		180		71.4%			
Carotid Intima–Media Thickness (mm)											
Left		0.66	±	0.19	0.60	0.95	±	0.21	1.00	0.000	^m^
Right		0.66	±	0.18	0.60	0.92	±	0.20	0.90	0.000	^m^
Albumin		4.2	±	0.5	4.3	4.0	±	0.6	4.1	0.000	^m^
Lymphocyte		2.1	±	0.7	2.1	2.1	±	0.9	2.1	0.650	^m^
CRP		3.2	±	9.9	1.1	9.5	±	26.8	2.9	0.000	^m^
CALLY Index		9.4	±	8.9	6.9	5.4	±	6.3	2.8	0.000	^m^
Department	Neurology	412		64.1%		100		39.7%		0.000	^X2^
	Cardiovascular Surgery	176		27.4%		118		46.8%		0.000	^X2^
	Other	50		7.8%		33		13.1%		0.014	^X2^

^m^ Mann–Whitney U test/^X2^ Chi-square test.

**Table 3:** Characteristics of Individuals Aged ≥ 65 Years by Carotid Artery Stenosis Status.

		Stenosis (−) (*n* = 127)				Stenosis (+) (*n* = 494)				*p*	
		Mean ± SD, *n* (%)			Median	Mean ± SD, *n* (%)			Median		
Age (years)		71.0	±	5.7	69.0	75.5	±	7.1	75.0	0.000	^m^
Sex	Female	64		50.4%		190		38.5%		0.015	^X2^
	Male	63		49.6%		304		61.5%			
Carotid Intima–Media Thickness (mm)											
Left		0.90	±	0.24	0.90	1.13	±	0.22	1.20	0.000	^m^
Right		0.87	±	0.22	0.90	1.09	±	0.22	1.10	0.000	^m^
Albumin		3.9	±	0.6	4.0	3.7	±	0.6	3.8	0.000	^m^
Lymphocyte		2.0	±	0.7	1.9	1.9	±	1.1	1.7	0.011	^m^
CRP		4.9	±	16.8	1.8	8.7	±	18.8	3.5	0.000	^m^
CALLY Index		6.0	±	6.3	3.8	3.5	±	4.6	1.9	0.000	^m^
Department	Neurology	57		44.9%		151		30.6%		0.002	^X2^
	Cardiovascular Surgery	54		42.5%		241		48.8%		0.207	^X2^
	Other	12		9.4%		98		19.8%		0.006	^X2^

^m^ Mann–Whitney U test/^X2^ Chi-square test.

**Table 4:** Comparison of Carotid Intima–Media Thickness and CALLY Index levels according to the degree of Carotid Artery Stenosis.

		Degree of Stenosis																
		^1^ Normal (*n*:770)			^2^ Mild (*n*:596)			^3^ Moderate (*n*:87)			^4^ Severe (*n*:53)			^5^ Occlusion (*n*:10)			*p*	
Carotid Intima–Media Thickness (mm)	Mean + SD	0.41	±	0.25	0.82	±	0.34	0.98	±	0.26	1.10	±	0.16	0.97	±	0.28	0.000 ^K^	^K^
CALLY Index	Mean + SD	8.86	±	8.62	4.52	±	5.55	2.97	±	4.45	2.32	±	3.08	0.93	±	0.75	0.000 ^K^	^K^
	Median	6.62			2.52 ^1^			1.23 ^12^			1.17 ^12^			0.67 ^12^				

^K^ Kruskal–Wallis.

**Table 5:** Association of carotid intima–media thickness (CIMT) with clinical characteristics.

Variables	Left CIMT (Mean ± SD)	*p*-Value	Right CIMT (Mean ± SD)	*p*-Value
**Sex**		0.020		0.016
Male	0.89 ± 0.29		0.87 ± 0.27	
Female	0.85 ± 0.28		0.84 ± 0.27	
**Age group**		<0.001		<0.001
<65 years	0.74 ± 0.23		0.73 ± 0.22	
≥65 years	1.08 ± 0.24		1.04 ± 0.23	
**Hypertension**		<0.001		<0.001
Present	0.96 ± 0.28		0.94 ± 0.27	
Absent	0.81 ± 0.27		0.79 ± 0.25	
**Diabetes mellitus**		<0.001		<0.001
Present	0.93 ± 0.28		0.91 ± 0.27	
Absent	0.86 ± 0.28		0.84 ± 0.27	
**Hyperlipidemia**		<0.001		<0.001
Present	0.95 ± 0.27		0.92 ± 0.26	
Absent	0.84 ± 0.29		0.83 ± 0.27	
**Smoking**		0.254		0.386
Present	0.86 ± 0.29		0.85 ± 0.27	
Absent	0.88 ± 0.29		0.86 ± 0.27	

**Table 6:** Association of baseline characteristics with carotid artery stenosis.

Parameters	Stenosis (+) (*n* = 746)	Stenosis (−) (*n* = 770)	*p*-Value
**Sex, n (%)**			<0.001
Male	484 (64.9)	430 (55.8)	
Female	262 (35.1)	340 (44.2)	
**Age (years)**	69.09 ± 11.25	50.68 ± 12.81	<0.001
**Hypertension, n (%)**	440 (59.0)	237 (30.8)	<0.001
**Diabetes mellitus, n (%)**	222 (29.8)	159 (20.6)	<0.001
**Hyperlipidemia, n (%)**	323 (43.3)	172 (22.3)	<0.001
**Smoking, n (%)**	231 (31.0)	246 (31.9)	0.680
**Albumin (g/dL)**	3.78 ± 0.62	4.18 ± 0.54	<0.001
**Lymphocyte count**	1.94 ± 1.01	2.12 ± 0.74	<0.001
**CRP**	8.97 ± 21.6	3.50 ± 11.4	<0.001
**CALLY index**	4.13 ± 5.31	8.86 ± 8.62	<0.001

**Table 7:** Multivariable logistic regression analysis for factors associated with carotid artery stenosis (overall).

Parameters	Beta	SE	*p*-Value	OR	95% CI for OR	
					Lower	Upper
CALLY index	−0.059	0.011	<0.001	0.943	0.923	0.964
Sex	0.608	0.143	<0.001	1.837	1.387	2.432
Age	0.108	0.006	<0.001	1.114	1.100	1.128
Hypertension	0.572	0.141	<0.001	1.772	1.345	2.334
Diabetes mellitus	0.069	0.160	0.666	1.071	0.783	1.466
Hyperlipidemia	0.794	0.147	<0.001	2.213	1.660	2.950
Smoking	0.205	0.148	0.166	1.228	0.919	1.640

**Table 8:** Multivariable logistic regression analysis for factors associated with carotid artery stenosis (age < 65).

Parameters	Beta	SE	*p*-Value	OR	95% CI for OR	
					Lower	Upper
CALLY index	−0.059	0.013	<0.001	0.942	0.918	0.967
Sex	0.653	0.191	0.001	1.920	1.320	2.794
Age	0.142	0.013	<0.001	1.152	1.124	1.182
Hypertension	0.623	0.188	0.001	1.864	1.290	2.694
Diabetes mellitus	0.197	0.224	0.381	1.217	0.784	1.890
Hyperlipidemia	0.751	0.189	<0.001	2.119	1.463	3.070
Smoking	0.187	0.188	0.322	1.205	0.833	1.743

**Table 9:** Multivariable logistic regression analysis for factors associated with carotid artery stenosis (age ≥ 65).

Parameters	Beta	SE	*p*-Value	OR	95% CI for OR	
					Lower	Upper
CALLY index	−0.063	0.020	0.001	0.939	0.904	0.975
Sex	0.492	0.218	0.024	1.636	1.066	2.510
Age	0.110	0.019	<0.001	1.117	1.075	1.160
Hypertension	0.631	0.218	0.004	1.880	1.226	2.883
Diabetes mellitus	0.027	0.228	0.906	1.027	0.658	1.605
Hyperlipidemia	0.787	0.238	0.001	2.197	1.378	3.502
Smoking	0.214	0.246	0.385	1.239	0.765	2.006

**Table 10:** Multivariable linear regression analysis of factors associated with carotid intima–media thickness (CIMT).

Variables	Left CIMT (β, SE)	*p*-Value	95% CI	Right CIMT (β, SE)	*p*-Value	95% CI
CALLY index	−0.007 (0.001)	<0.001	−0.009 to −0.006	−0.007 (0.001)	<0.001	−0.008 to −0.005
Age	0.011 (<0.001)	<0.001	0.011 to 0.012	0.010 (<0.001)	<0.001	0.009 to 0.011
Sex	0.025 (0.010)	0.019	0.004 to 0.045	0.025 (0.010)	0.014	0.005 to 0.046
Hypertension	0.029 (0.011)	0.009	0.007 to 0.050	0.028 (0.011)	0.010	0.007 to 0.048
Hyperlipidemia	0.031 (0.011)	0.005	0.010 to 0.053	0.019 (0.011)	0.077	−0.002 to 0.041
Diabetes mellitus	0.002 (0.012)	0.852	−0.021 to 0.026	0.000 (0.012)	0.985	−0.023 to 0.023
Smoking	0.004 (0.011)	0.717	−0.018 to 0.026	0.008 (0.011)	0.477	−0.014 to 0.029
Albumin level	−0.009 (0.010)	0.372	−0.027 to 0.010	−0.021 (0.009)	0.025	−0.039 to −0.003
Lymphocyte count	0.018 (0.006)	0.004	0.006 to 0.030	0.020 (0.006)	0.001	0.008 to 0.032
CRP	0.002 (<0.001)	<0.001	0.001 to 0.002	0.001 (<0.001)	<0.001	0.001 to 0.002

## Data Availability

The data presented in this study are available on reasonable request from the corresponding author. The data are not publicly available due to patient privacy and institutional ethical restrictions.

## Abbreviations

The following abbreviations are used in this manuscript:

CALLY	C-reactive protein–albumin–lymphocyte index
CRP	C-reactive protein
CIMT	Carotid intima–media thickness
US	Ultrasonography
ROC	Receiver operating characteristic
AUC	Area under the curve
CI	Confidence interval
OR	Odds ratio
SD	Standard deviation
SE	Standard error
SPSS	Statistical Package for the Social Sciences
